# Multiplexed Dark FRET Biosensors: An accessible live-cell platform for target- and cell-specific monitoring of protein-protein interactions in 2D and 3D model systems

**DOI:** 10.21203/rs.3.rs-6580769/v1

**Published:** 2025-05-12

**Authors:** Anthony Braun, Elly Liao, Nagamani Vunnam, Marguerite Murray, Jonathan Sachs

**Affiliations:** University of Minnesota; University of Minnesota; University of Minnesota; University of Minnesota; University of Minnesota

**Keywords:** Multiplexed FRET, Target-Specificity, High-throughput screening, 3D spheroid

## Abstract

Simultaneously monitoring multiple protein-protein interactions in live cells remains a key challenge in biology and drug discovery. While multiplexed FRET enables parallel molecular readouts, existing approaches are often constrained by spectral overlap, complex instrumentation, or incompatibility with live-cell models. To overcome these limitations and increase accessibility to the broader biological community, we present Multiplexed Dark FRET (MDF), a genetically encoded platform that uses spectrally distinct donors (mNeonGreen, mScarlet-I3) paired with non-emissive acceptors (ShadowY, ShadowR). Using fluorescence lifetime detection, we demonstrate MDF’s versatility through three biologically and translationally relevant examples: (1) cell-type specific biosensing in organoids, as exemplified in 3D neuro–glial spheroids; (2) target specificity for drug discovery, through discrimination of TNFR1 versus TNFR2 receptor conformations; and (3) protein misfolding, as exemplified through simultaneous monitoring of alpha-synuclein oligomerization and misfolding. MDF provides a scalable framework for real-time, live-cell biosensing across high-throughput, target-specific, and tissue-level applications in complex biological systems.

## INTRODUCTION

Fluorescent proteins are foundational tools in modern biology used to track protein expression and subcellular localization and to monitor cellular processes across diverse systems. From single-molecule assays to whole-organism imaging, genetically encoded fluorophores have revolutionized how biologists interrogate the living cell. Multiplexed imaging using multiple fluorescent proteins — each with distinct excitation and emission properties — is now routine for studying colocalization, lineage tracing, or parallel signaling events.

Förster resonance energy transfer (FRET) builds upon this foundation, enabling real-time monitoring of protein–protein interactions, conformational changes, and dynamic signaling in living cells. FRET biosensors have been widely adopted using fluorescence intensity or fluorescence lifetime (FLT) detection modalities. However, expanding FRET to track multiple interactions simultaneously presents significant challenges. Multiplexed FRET systems must navigate crowded emission spectra, complex spectral unmixing, and the need for carefully balanced controls to distinguish overlapping signals. While advanced multiplexing approaches — such as PIE-FLIM^1,2^, dual-color fluorescence cross-correlation spectroscopy^3^, donor photochromism^4^, and FRETfluor barcoding^5^ — have ingeniously addressed some of these barriers, they often require specialized optics, complex reagent preparations, or are restricted to *in vitro*^6–8^ or fixed samples^9^, limiting their general accessibility to the broader research community.

For the first time, we show the power and broad utility of multiplexing FRET using spectrally distinct donors — mNeonGreen (mNg) and mScarlet-I3 (mScI3) — with recently developed non-emissive dark acceptors — e.g., ShadowY^10^ (ShY) and ShadowR^11^ (ShR) — for biological application. This genetically encoded platform expands the accessibility of multiplexed biosensing of protein-protein interactions in living cells and is herein called Multiplexed Dark FRET (MDF). MDF enables orthogonal FRET readouts from donor fluorescence alone, significantly reducing spectral crosstalk and simplifying experimental design via straightforward implementation using standard FLT or intensity-based imaging systems. Despite the demonstrated advantages of dark acceptors, their use in simultaneous, genetically encoded live-cell biosensing has remained unexplored with only few studies leveraging them for single channel FRET or FLIM applications^12–14^. MDF fills this gap by expanding the accessibility of multiplexed FRET measurements for real-time analysis of protein–protein interactions in both 2D and 3D biological systems.

Our previous work has established a broad suite of FRET biosensors that monitor protein-protein interactions and conformation changes for high-throughput screening (HTS). These biosensors include several tumor necrosis factor superfamily receptors (TNFSFRs; e.g., TNFR1, TNFR2, and DR5^15–21^) and multiple amyloidogenic proteins (e.g., alpha-synuclein [aSyn], tau, TDP43, and Huntington protein)^22–26^.

We demonstrate the versatility of MDF through three biologically relevant applications. First, we apply MDF in neuron–microglia spheroids to enable spatially resolved, cell-type-specific biosensing within tissue-like environments—addressing a critical need for dynamic, multiplexed measurements in advanced 3D systems. Second, we show that MDF allows live-cell discrimination of TNFR1 and TNFR2 conformational responses to pharmacologic agents, providing a streamlined and scalable approach for assessing receptor-selective activity in anti-TNFR drug discovery. Third, we leverage MDF to simultaneously monitor alpha-synuclein oligomerization and intramolecular misfolding, delivering orthogonal insight into distinct steps of the amyloidogenic cascade relevant to synucleinopathies. Together, these applications position MDF as a powerful, accessible platform for multiplexed monitoring of protein interactions and signaling in both HTS and physiologically complex biological systems.

## RESULTS

### Dark acceptor-based biosensors enable orthogonal FRET in live cells

**Figure 1:**
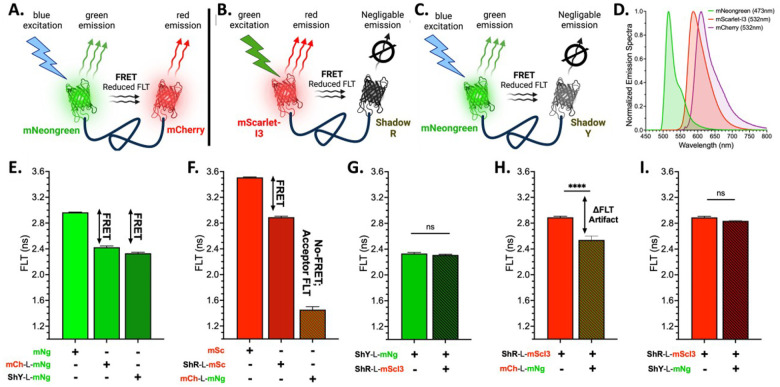
Multiplexed Dark FRET with Shadow Acceptors reduces spectral crosstalk and improves signal resolution. Control FRET biosensors are designed with donor and acceptor fused with a 32-amino acid G_4_S linker with a conventional FRET biosensor (A. mNg/mCh) and shadow multiplexed FRET biosensors (B. mNg/ShY and C. mScI3/ShR). D. Emission spectra for mNg (G/R donor), mCh (G/R acceptor) and mScI3 (R/FR donor) highlights the spectral overlap for the donor and acceptor fluorophores which compound multiplexing FRET systems. FRET is determined by monitoring changes in FLT with and without acceptor with E. demonstrating the green donor channel’s FLT (473nm excitation FLT and F. highlighting the red donor channel (532nm excitation). The change in FLT (ΔFLT) relative to donor only for donor/acceptor systems indicates FRET. However, the acceptor emission from mCh-L-mNg results in a short FLT signal in the red channel, compounding direct measure of FRET for a red donor biosensor. G. Monitoring red channel FLT for mixed biosensor samples with cells expressing either ShR-L-mScI3 or mCh-L-mNg constructs results in a significantly reduced FLT observation (non-real FRET signal). H. When two MDF shadow acceptor biosensors are used (ShY-L-mNg and ShR-L-mScI3) this artifact is not present, allowing the recovery of each individual population’s FLT. I. Due to the lack of interfering fluorophores, the FLT artifact does not manifest in the blue-shifted FRET pair under multiplexed FRET conditions. Panels A-C generated in Biorender.

We engineered a series of donor-only and donor–acceptor FRET constructs (Fig. 1A–C) to illustrate the technical challenges of multiplexing FRET biosensors and to showcase how the MDF platform overcomes these limitations. These biosensors include a conventional FRET pair (mNg/mCh) and two dark acceptor FRET pairs (mNg/ShY, mScI3/ShR; **Supplemental Figure S1**). Positive FRET controls were constructed following an acceptor-linker-donor structure with a 32 amino-acid GSG_4_ linker segment. All dual-fluorophore biosensors were engineered with the acceptor at the N-terminus to improve expression fidelity and minimize artifacts from incomplete fluorophore maturation. N-terminal fusion proteins often fold and express more reliably than C-terminal fusions^27–29^. By placing the acceptor at the N-terminus, we minimize misfolded or truncated products that would artifactually lower FRET efficiency.

Our FLT-plate reader has two laser lines, a 473nm laser exciting mNg and 532nm laser for mCh and mScI3. Figure 1D illustrates the emission spectra for the three conventional XFPs used in this study. Fluorescent micrographs for donor only and positive FRET acceptor-linker-donor biosensors expressed in HEK293T cells are presented in **Supplemental Figure S2**. Only the mCh-L-mNg biosensor results in overlapped red/green signal due to emission from both donor and acceptor fluorophores. Using FLT based FRET allows for monitoring of donor emission alone, which for mNg occurs over a sparse region of the spectrum. In contrast, both mCh and mScI3 emission spectra span the same wavelengths. Figure 1E highlights that both mNg/mCh and mNg/ShY biosensors have a robust change in FLT (ΔFLT) relative to mNg only condition, which indicates FRET. A similar ΔFLT is observed for the mScI3/ShR FRET pair relative to mScI3 alone (Fig. 1F).

When multiplexing the conventional FRET (mNg/mCh) biosensor with a red dark-acceptor (mScI3/ShR) biosensor, the emission overlap between mCh and mScI3 — acceptor and donor XFPs for separate biosensors — confounds the red channel’s FLT measurement (Fig. 1G). The resulting observed reduction in FLT can be easily misinterpreted as FRET, when it is actually a false positive response emanating from the convolution of mCh and mScI3’s FLT. However, when we mix two cell populations that express the two MDF biosensors (mNg/ShY; mScI3/ShR), we eliminate the spectral overlap and are able to isolate each specific FLT signal for both the red (Fig. 1H) and green (Fig. 1I) channels with no significant differences between single or mixed cell conditions.

### MDF Application #1: Cell-type-specific FRET readouts in 3D neuro-glial spheroids

Recent advances in genetically encoded fluorescent biosensors have enabled the investigation of dynamic signaling, differentiation, and disease processes within 3D organoid systems. As one of many examples, calcium indicators like GCaMP have been applied across brain, cardiac, and intestinal organoids to track functional activity and tissue maturation^30^, while FRET-based biosensors for kinases such as ERK, PKC, and PKA have been adapted to monitor spatially resolved intracellular signaling in response to environmental cues and therapeutic agents^31–33^. These tools are increasingly paired with advanced imaging modalities—such as light-sheet microscopy for volumetric acquisition and fluorescence lifetime imaging microscopy (FLIM) for quantitative FRET detection in optically dense tissues^30,34^. However, current applications rely on single-channel reporters or require sequential measurements, limiting the ability to resolve multiple concurrent signaling events. As organoids and assembloids grow in complexity—with multiple interacting cell types and spatial organization—there is a growing need for biosensing strategies that can interrogate multiple protein interactions or signaling dynamics in a cell-type-specific manner. MDF meets this challenge by enabling orthogonal FRET readouts from genetically encoded biosensors expressed in distinct cellular compartments within live 3D systems.

**Figure 2:**
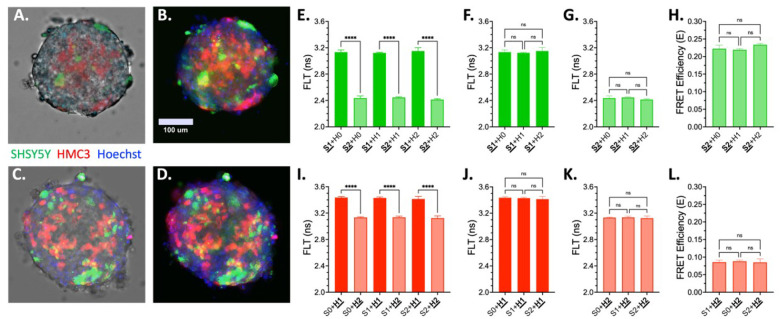
Multiplexed MDF biosensors implemented in 3D neuro-glial spheroids (NGS) resolves cell-type specific FRET. Control MDF biosensors were expressed in a series of SHSY5Y/HMC3 spheroids. Green: S0 = naive SHSY5Y; S1 = mNg; S2 = ShY-L-mNg; Red: H0 = naïve HMC3; H1 = mScI3; H2 = ShR-L-mScI3. A-D. Live-cell fluorescent imaging of two distinct S1+H1 NGS with green SHSY5Y derived neurons and red HMC3 microglia. Transmitted light overlays in A. and C. highlights NGS perimeter. Cell nuclei are stained with Hoechst dye (blue). E. Comparison of SHSY5Y FLT in NGS spanning all HMC3 biosensor conditions. F. No significant difference between SHSY5Y donor only (S1) across spheroid type. G. No significant difference between SHSY5Y donor + acceptor (S2) across all NGS compositions. H. The resulting green channel FRET is not changed across all NGS compositions. I. Comparison of HMC3 FLT in spheroids spanning all SHSY5Y biosensor conditions. J. No significant difference between HMC3 donor only (H1) across NGS. K. No significant difference between HMC3 donor + acceptor (H2) across all spheroid compositions. L. The resulting red channel FRET is not changed across all NGS compositions.

To evaluate the utility of MDF in tissue-like systems, we applied the platform to 3D self-assembled aggregates composed of SHSY5Y-derived neurons and HMC3 microglia^35^. These co-culture neuro-glial spheroids (NGS) offer a physiologically relevant model for studying intercellular signaling. SHSY5Y (S) and HMC3 (H) cells were transfected with either donor-only (mNg or mScI3) or donor-acceptor constructs (ShY-L-mNg or ShR-L-mScI3), then self-assembled into NGS using a combination of untransfected (S0, H0) or transfected (S1, H1 for donor only; S2, H2 for donor-acceptor) cell populations. Figure 2A–D present live cell images of two S2 + H2 NGS. FLT analysis showed robust FRET responses within each cell type, with donor–acceptor constructs exhibiting significantly reduced lifetimes compared to donor-only controls (Fig. 2E, 2I). Critically, the presence of biosensors in one cell type had no impact on FRET detection in the other, confirming that MDF maintains signal specificity in 3D mixed-cell spheroids (Fig. 2F–H, 2J–L).

The transient transfection of SHSY5Y and HMC3 cells typically yields modest efficiency (~ 30%), resulting in a fraction of non-transfected cells within the NGS. Despite this, both green and red donor FLT signals were readily detectable, demonstrating that even partial biosensor expression is sufficient for signal acquisition.

### MDF Application #2: Multiplexed FRET differentiates TNFR1 and TNFR2 conformation changes in live cells

We have previously demonstrated that cellular TNFSFR FRET biosensors report a basal FRET signal from pre-ligand dimers that reflects the proximity of the intracellular receptor termini and serves as a sensitive proxy for receptor conformation^18,19,21,36^. Importantly, we observed that perturbations to this basal FRET—through mutagenesis or small-molecule treatment — correlate with downstream signaling outcomes, including IκB degradation and activation of NF-κB transcription factors^16,17,21^. This conserved conformational signature across TNFSFRs provides a powerful foundation for HTS of receptor-specific modulators using FRET-based readouts.

We applied the MDF platform to extend this approach to a dual-receptor format, enabling simultaneous monitoring of TNFR1 and TNFR2 in live cells. Although these receptors bind the same ligand — TNF — they drive distinct and often opposing signaling outcomes: TNFR1 primarily mediates pro-inflammatory and apoptotic pathways, while TNFR2 promotes tissue regeneration and neuroprotection^37–41^. Despite this dichotomy, current anti-TNF therapies indiscriminately neutralize TNF itself — suppressing both beneficial and detrimental signaling arms — which has led to well-documented adverse effects including increased risk of infection, demyelinating disease, and impaired tissue repair^41–43^. As a result, there is increasing interest in therapeutic approaches that selectively modulate receptor activity without altering ligand availability. However, the high sequence and structural similarity between TNFR1 and TNFR2 complicates the identification of receptor-selective compounds, often requiring additional validation assays beyond the primary screen. Conventional drug screening workflows often require orthogonal, time- and resource-intensive secondary assays to distinguish on-target effects^16,20,44,45^. A biosensing platform that can resolve receptor-specific activity in real time — within a single assay — is thus a powerful tool for advancing TNFSFR-targeted drug discovery.

**Figure 3:**
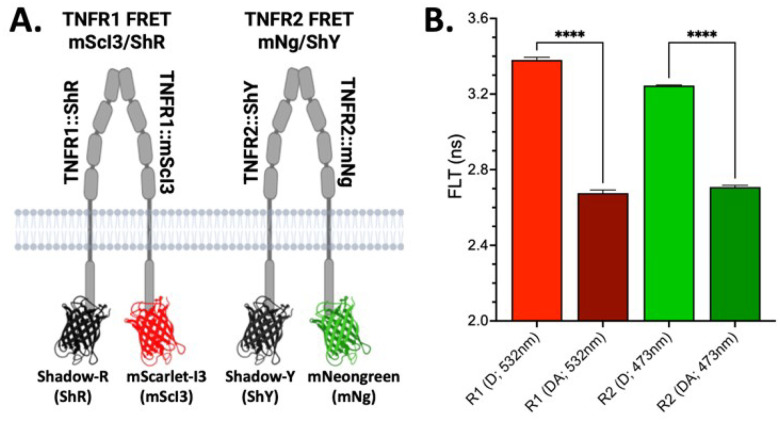
Design of receptor specific MDF for TNFRSF targets. A. Schematic of TNFR1 (R1 *left*; mScI3/ShR fusion) and TNFR2 (R2 *right*; mNg/ShY fusion) FRET biosensors using the MDF FRET pairs. Both receptors are full-length, functional constructs with a C-terminal fusion. B. Expression of donor only (D) or donor and acceptor (DA; 1:1 ratio) result in a robust ΔFLT corresponding to FRET. Panel A generated in Biorender.

**Figure 4:**
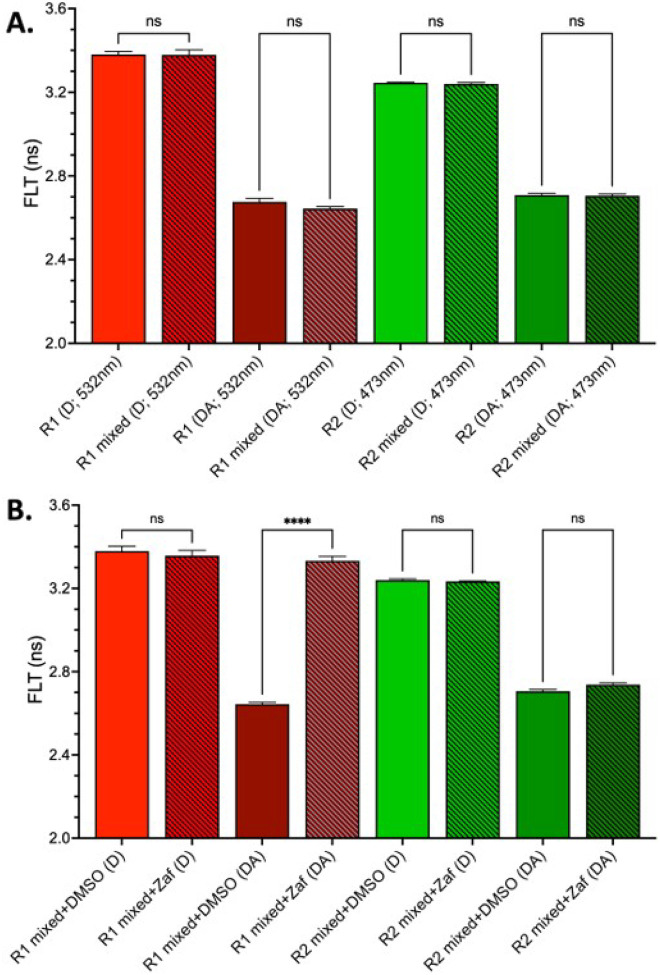
MDF differentiates receptor specific TNFR1 and TNFR2 conformation changes in mixed cell populations. A. R1 and R2 MDF biosensors have no change in FLT for single or mixed samples for both D and DA biosensors. B. Using the R1 and R2 mixed MDF biosensor system the TNFR1 small molecule inhibitor Zafirlukast (Zaf) disrupts R1 ΔFLT while having no change in R2 ΔFLT.

To address this challenge, we developed full-length, conformationally responsive FRET biosensors for TNFR1 and TNFR2 with C-terminal fusions of mScI3/ShR (TNFR1) pair and mNg/ShY (TNFR2), see Fig. 3A. These biosensors report on ligand-independent conformational changes within PLAD-mediated receptor dimers. Transient co-transfection of TNFR1 and TNFR2 biosensors in HEK293T cells at a 1:1 donor-to-acceptor ratio resulted in red and green puncta across the plasma membrane (**Supplemental Figure S3**) and a robust FRET signal (Fig. 3B). When combined in mixed cell populations, MDF preserved channel specificity, allowing for orthogonal monitoring of each receptor’s conformation (Fig. 4A).

To test whether MDF could distinguish receptor-specific perturbations, we treated biosensor-expressing cells with zafirlukast, a compound that we previously identified as a TNFR1-selective allosteric inhibitor^16^. Zafirlukast treatment abolished the basal FRET signal in the TNFR1 biosensor while having no measurable effect on TNFR2 (Fig. 4B), confirming selective engagement and the capacity of MDF to resolve receptor-specific drug responses. These findings establish MDF as a high-resolution platform for multiplexed biosensing of TNFSFRs and demonstrate its utility in identifying conformation-selective, receptor-specific modulators with immediate translational relevance.

### MDF Application #3: Dual aSyn biosensors monitor aggregation and misfolding

Alpha-synuclein misfolding and aggregation is a hallmark of Parkinson’s disease, progressing through a multi-step cascade from monomeric aSyn to toxic oligomers and eventually to fibrillar inclusions^46–52^. Pinpointing when and how these aggregation events become pathogenic remains a major challenge. Our previous work established two complementary FLT-FRET biosensors to monitor distinct aspects of aSyn aggregation. One biosensor detects intermolecular FRET between monomers, each fused at the C-terminus to either a donor or acceptor fluorophore, enabling quantification of oligomerization^24^. The second employs an intramolecular dual-fusion design, with donor and acceptor fluorophores positioned at the N- and C-termini of the same aSyn molecule, allowing detection of both oligomerization and conformation changes^24,25^. These biosensors have been successfully applied in high-throughput screening campaigns, leading to the identification of nanomolar-potency inhibitors that suppress aSyn-induced toxicity^24,25^.

**Figure 5:**
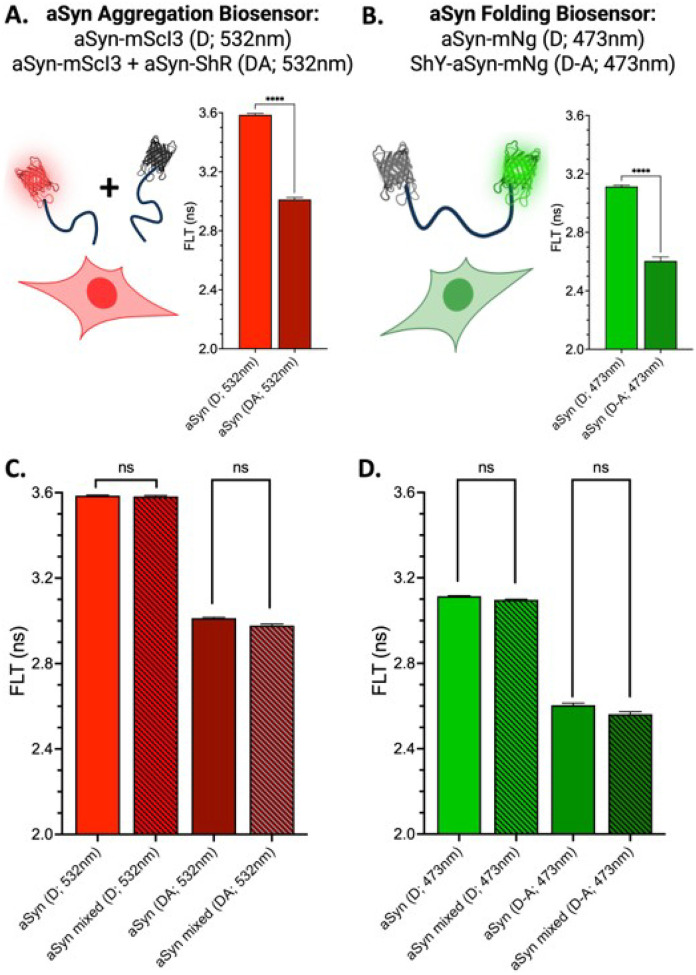
MDF enables concurrent monitoring of two mechanistically different aSyn aggregation biosensors. A. Schematic of aSyn-mScI3/aSyn-ShR single-fusion biosensor and corresponding FLT for donor only and donor-acceptor (1:4 D:A ratio) conditions. B. Schematic of ShY-aSyn-mNg double fusion biosensor and corresponding FLT for donor only and donor-acceptor conditions. aSyn FLT-FRET biosensors under single and mixed cell biosensor conditions highlighting no difference between monitoring aggregation biosensor (C) or folding biosensor (D). Panels A-B generated in Biorender.

The MDF platform extends our previously developed aSyn biosensors by enabling their simultaneous use in a multiplexed configuration. This dual-sensor approach allows real-time tracking of two mechanistically distinct events: oligomer assembly, which is reported by both biosensors, and monomer misfolding, which is uniquely captured by the intramolecular sensor through changes in FRET that are unique to the double fusion biosensor (i.e., non-overlapping hit compounds across the two, intramolecular and intermolecular, biosensors). This setup provides an integrated view of aggregation state dynamics and allows discernment between interventions that target assembly, structural conformation, or both. Figure 5A illustrates the single-fusion aSyn biosensors (aSyn-mScI3 and aSyn-ShR), which report oligomerization via reduced donor fluorescence lifetime (FLT) when co-expressed at a 1:4 donor-to-acceptor ratio^24^. Figure 5B shows the dual-fusion aSyn sensor (ShY-aSyn-mNg), which detects both folding and oligomerization via FLT reduction relative to donor-only controls^25^. Transient expression of these MDF biosensors (**Supplemental Figure S4**) results in diffuse cytoplasmic distribution, consistent with our previous studies^24,25^. As with the TNFR1/TNFR2 system, FLT measurements can be acquired under single or mixed-cell conditions (Figs. 5C and 5D), enabling simultaneous screening of both biosensors. Importantly, using the same target protein across two spectrally distinct channels enhances the robustness of the assay by reducing susceptibility to false positives from fluorescent interfering compounds — an essential advantage for HTS applications.

## DISCUSSION

MDF democratizes live-cell multiplexed FRET biosensing by reducing experimental complexity, minimizing spectral crosstalk, and eliminating the need for chemical labeling or specialized instrumentation. By pairing spectrally distinct donors with non-emissive dark acceptors, MDF enables orthogonal detection of two protein interactions using standard fluorescence lifetime (FLT) or intensity-based systems. Unlike more technically demanding multiplexed FRET strategies such as PIE-FLIM^1,2^, dual-color fluorescence cross-correlation spectroscopy^3^, or FRETfluor barcoding^5^, MDF operates with accessible hardware and workflows, positioning it as a scalable, genetically encoded tool for both HTS and emerging 3D model systems.

We validated MDF across three biological applications, each highlighting a unique advantage of the platform. First, in 3D NGSs, MDF biosensors enabled cell-type specific, spatially resolved FRET readouts. Even with modest transfection efficiencies, MDF provided robust, channel-specific FRET signals, enabling the study of protein interactions in defined cell populations. For HTS, population-level changes in FLT offer powerful readouts of global pathway activity or aggregation. Expanding MDF to spatially resolved FLIM would further reveal heterogeneity across microenvironments. Importantly, mixed populations of biosensor-positive and -negative cells enable studies of intercellular communication, signal propagation, and tissue architecture, supporting applications such as bioprinting or modeling neuroinflammatory cascades in disease-relevant 3D systems.

Second, we applied MDF to dissect receptor-specific conformational changes in TNFR1 and TNFR2. Simultaneous tracking of both receptors revealed selective inhibition of TNFR1 by zafirlukast, demonstrating the platform’s ability to resolve receptor specificity in real time. This eliminates the need for sequential secondary assays and addresses a key challenge in anti-TNF therapeutic development.

Third, MDF was used to probe mechanistically distinct steps in aSyn aggregation. By combining intermolecular and intramolecular FRET biosensors, MDF enables simultaneous detection of oligomerization and conformational misfolding — key features of synucleinopathies. This dual-readout approach improves mechanistic insight and assay robustness, allowing researchers to differentiate between compounds that affect oligomer formation versus conformational collapse. Additionally, pairing the same biosensor design in both channels offer internal target redundancy that reduces false positives from fluorescent compound interference.

MDF has broad applications across biological systems where multiple protein interactions underlie complex signaling and functional outcomes. As one of many examples, MDF holds particular promise for investigating co-proteinopathies in neurodegenerative diseases, where concomitant aggregation of proteins such as aSyn, tau, TDP43, and Aβ is recognized as a driver of disease pathology^53–61^. Recent studies demonstrate that tau and aSyn can cross-seed aggregation, exacerbating neurotoxicity^53–57^. Furthermore, the presence of TDP43 pathology in a substantial fraction of Alzheimer’s disease cases^59–61^ correlates with worsened cognitive decline. Understanding how these misfolded species interact in real time within live-cell or 3D models will be essential for unraveling disease mechanisms. For example, one application of MDF could involve expressing an aSyn-targeted (green channel) and tau-targeted (red channel) biosensor in distinct neuronal populations within NGS. This configuration would allow researchers to assess how shared cellular stressors influence aggregation dynamics of each protein independently, and to identify both common and protein-specific therapeutic responses in a spatially defined, multicellular context.

Biomolecular condensates — e.g., stress granules, p-bodies — are formed through liquid–liquid phase separation and represent another class of dynamic, multicomponent assemblies. These membraneless organelles rely on multivalent protein–RNA interactions and are governed by intrinsically disordered domains^62^. Emerging work highlights their roles in transcription, stress response, and disease, and calls for tools that can simultaneously track multiple protein assemblies within or across condensates^63–68^. MDF provides a genetically encoded, multiplexed platform ideally suited to dissect how these multicomponent assemblies form, dissolve, and influence disease pathogenesis.

MDF’s multiplexed capabilities are also well suited to interrogating immune signaling cascades in neurobiology and oncology. In neuron–glia co-cultures or brain assembloids, for example, one biosensor could track neuronal stress or receptor activation while the other reports glial cytokine responses or inflammatory transcriptional events. This level of spatially compartmentalized signaling is essential for dissecting cell-specific neuroinflammatory cascades in neurodegenerative disease models. Recent studies emphasize the need for simultaneous imaging of cytokine and neuronal signals in 3D systems to understand how inflammation spreads through tissue-level networks^69,70^. In the context of CAR-T cell therapy, MDF can provide real-time dual readouts of antigen receptor activation and effector function. This pairing enables mechanistic studies linking signal input with therapeutic output. Multiplexed live-cell reporters are increasingly recognized as essential tools for optimizing CAR construct design and reducing toxicity, bridging signaling features with function in tumor microenvironments^71,72^. MDF provides a scalable solution for these high-content screening applications.

While MDF represents a major advancement for live-cell multiplexed biosensing, several limitations remain that will guide future development. Current MDF implementation relies on FLT, which — though increasingly available — is less ubiquitous than intensity-based platforms. Future optimization for ratiometric or intensity-only readouts could enable broader deployment in multimode plate readers and flow cytometers, enhancing the accessibility of multiplexed FRET assays for therapeutic discovery and translational applications. In complex 3D models, challenges include biosensor delivery, expression control, and imaging depth. Viral vectors or stable integration will be critical for consistent biosensor expression, especially in iPSC-derived organoids. Inducible or cell-specific promoters (e.g., tetracycline- or Cre-lox–regulated systems) will help mitigate developmental perturbations^73–75^. Imaging larger organoids will require advanced modalities such as two-photon FLIM or light-sheet microscopy^76–78^, and the impact of spheroid size on FLT signal must be carefully addressed in future work.

Despite these limitations, MDF transforms previously inaccessible FRET experiments into practical, high-content assays suitable for both basic and translational research. As the need grows for real-time, multiplexed monitoring of molecular interactions in live systems, MDF provides an accessible and scalable solution for probing dynamic signaling and protein-protein interactions in physiologically relevant models.

## Supplemental Information:

*Materials and Methods*, one Supplemental Table and four Supplemental Figures.

Supplementary Files

This is a list of supplementary files associated with this preprint. Click to download.

- BraunARSIMultiplexFRET.pdf
